# Heritable pulmonary arterial hypertension: new genetic findings and environmental triggers

**DOI:** 10.1038/s41598-025-34167-0

**Published:** 2026-01-29

**Authors:** Memoona Shaukat, Ekkehard Grünig, Simon Haas, Jan Haas, Mohammad Panahi, Martin Granzow, Tobias J. Lange, Stefan Stadler, Natascha Sommer, Peter Dorfmüller, Benjamin Meder, Satenik Harutyunova, Benjamin Egenlauf, Panagiota Xanthouli, Katrin Hinderhofer, Christina A. Eichstaedt

**Affiliations:** 1 Center for Pulmonary Hypertension, Thoraxklinik Heidelberg gGmbH at Heidelberg University Hospital and Translational Lung Research Center Heidelberg (TLRC), German Center for Lung Research (DZL), Heidelberg, Germany; 2 Laboratory for Molecular Genetic Diagnostics, Institute of Human Genetics, Heidelberg University, Heidelberg, Germany; 3 Institute for Cardiomyopathies Heidelberg (ICH) and Center for Cardiogenetics, Heidelberg University Hospital, and DZHK, Site Heidelberg/Mannheim, Heidelberg, Germany; 4 Department of Pneumology, Kliniken Südostbayern AG, Kreisklinik Bad Reichenhall, Bad Reichenhall, Germany; 5 Department of Internal Medicine II, University Medical Center Regensburg, Regensburg, Germany; 6 Medical and Policlinic II, Excellence Cluster Cardio-Pulmonary Institute (CPI), Member of the German Center for Lung Research (DZL), Justus-Liebig University Giessen, Giessen, Germany; 7 Institute for Lung Health (ILH), Giessen, University Hospital of Gießen and Marburg, Universities of Giessen and Marburg Lung Center (UGMLC), German Center for Lung Research (DZL), Gießen, Germany; 8 Department of Internal Medicine III, Precision Digital Health, University of Heidelberg and Informatics for Life, and German Center for Cardiovascular Research (DZHK), Heidelberg, Germany; 9 Division of Rheumatology, Department of Internal Medicine V: Hematology, Oncology and Rheumatology, University Hospital Heidelberg, Heidelberg, Germany

**Keywords:** Pulmonary arterial hypertension, Transforming growth factor beta, Whole exome sequencing, Drug and toxin exposures, Diseases, Genetics, Medical research

## Abstract

Our goal was to identify new environmental or genetic causes in heritable pulmonary arterial hypertension (HPAH) families outside the 18 known diagnostics PAH genes. PAH gene panel sequencing was performed for 47 HPAH families which revealed pathogenic variants in 39 families. Five of the remaining families agreed to whole exome sequencing and to fill in a drug and toxin exposure questionnaire. In Family 1 and 2, mother and daughter with HPAH carried a likely pathogenic variant in the *CYBA* gene and a variant of uncertain significance in the *FKBP1A* gene, respectively, following ACMG guidelines. In Family 3, we detected a likely pathogenic variant in the *PTGR2* gene. These genes could play part in PAH pathogenesis but further functional analyses are required to corroborate these findings. In the remaining two families, we could not identify any plausible genetic cause. However, a father and son with PAH reported exposure to trichloroethylene, asbestos and tramadol in Family 4. In Family 5, two brothers with pulmonary veno-occlusive disease showed occupational toxin exposure. Thus, our findings indicate that not only a genetic predisposition but also environmental triggers should be investigated for HPAH patients.

**Supplementary Information:**

The online version contains supplementary material available at 10.1038/s41598-025-34167-0.

## Introduction

Pulmonary arterial hypertension (PAH) is a rare disease that affects small pulmonary vessels and leads to reduced exercise capacity, quality of life and ultimately causes right heart failure^1^. PAH can be classified as heritable PAH (HPAH) if a disease-causing genetic variant is detected or if multiple family members are affected^2^. The most common genetic cause of HPAH is a pathogenic variant in the bone morphogenetic protein receptor type 2 (*BMPR2*) gene, encoding a type 2 receptor of transforming growth factor beta (TGF-β) signaling pathway^3^. Pathogenic variants have also been discovered in further genes within and outside the BMPR2/TGF-β pathway^4^. All known 18 PAH genes can be tested at once in a cost-effective manner by gene panel sequencing based on next generation sequencing^5^. However, in 17% of HPAH patients no disease-causing variant in the established PAH genes can be identified despite comprehensive genetic testing^5^. This highlights the gaps in our knowledge about disease pathogenesis and the further need to detect novel genes, pathways, and triggers involved in the disease development. Whole exome sequencing (WES) or whole genome sequencing, can be a tool for the discovery of novel PAH genes and eventually provide better insight into the complex disease etiology to facilitate new therapeutic targets and potentially better treatment in future.

Beyond genetics causes, PAH can also be induced by environmental factors such as drugs or toxins^6^. Various drugs including appetite suppressants, alkylating agents, anti-tumor drugs or opioids have been identified as a trigger for PAH and pulmonary veno-occlusive disease (PVOD)^1^. Equally, environmental toxins such as organic solvents or toxic rapeseed oil are possible triggers of PAH or PVOD^6–9^.

In this study, we employed a holistic approach to identify the specific disease cause in PAH families. We started with PAH gene panel sequencing to search for pathogenic variants in 18 known PAH genes, WES to find rare deleterious variants in potentially novel PAH candidate genes in panel negative families, and finally investigated environmental factors that could have triggered disease development using a detailed medical and occupational history. For this purpose, we developed a special questionnaire for patients and their relatives to identify previous drug and toxin exposures to any substances listed as definite or possible associated with PAH in the ESC/ERS pulmonary hypertension (PH) guidelines and other literature.

## Methods

### Recruitment and clinical assessment of HPAH families

We enrolled 47 HPAH families from 2017 to 2020 at the Center for Pulmonary Hypertension at Thoraxklinik at Heidelberg University Hospital and further PH centers across Germany, including PH expert centers at the University Medical Center Regensburg, Charité University Medicine, University Hospital Leipzig, University Hospital Dresden, Cologne University Heart Center, University Hospital of Gießen and Marburg University of Greifswald, University Medical Center Hamburg-Eppendorf and Hannover Medical School, as reported previously^5^. Each HPAH family was characterized by at least having two affected family members with manifest PAH, who were diagnosed by right heart catheterization and clinically evaluated following the respective guidelines^1,10,11^. Additional clinical parameters included 6-minute walking distance (6MWD), World Health Organization (WHO) functional class, N-terminal pro b-type natriuretic peptide (NT-proBNP) and blood gas analysis measurements including diffusion capacity of carbon monoxide (DLCO). If an index patient reported PAH in additional deceased family members and no medical data were available at the time of the study, PAH was categorized as “suspected”. Family members provided written informed consent to be enrolled for genetic diagnostic and research testing. In 39 of these families a disease-causing variant was identified using next generation sequencing with a PAH-specific gene panel as reported previously^5^. The remaining families with no (likely) pathogenic variants in known PAH genes were approached to offer WES to patients and, if available, healthy family members. The ethics committee of the Medical Faculty of Heidelberg, Germany had no objections against this study and approved the experimental protocol (internal ID: S-925/2020). The study has been carried out in accordance with the Declaration of Helsinki for medical research involving human subjects. Pedigrees were recorded for families (Genopro, Genogram Software, Hong Kong).

### Sample collection and gene panel sequencing

EDTA blood samples were automatically extracted (Autopure or QIAsymphony, Qiagen, Germany). Next generation panel sequencing was carried out on our PAH-specific gene panel including 16–18 PAH diagnostics and > 40 candidate genes (European patent ID: EP3507380; https://register.epo.org/application?number=EP17762088), as previously reported^5^. Diagnostic genes included *ABCC8*,* AQP1*,* ACVRL1*,* ATP13A3*,* BMPR1B*,* BMPR2*,* CAV1*,* EIF2AK4*,* ENG*,* GDF2*,* KCNA5*,* KCNK3*,* KDR*,* KLF2*,* SMAD4*,* SMAD9*,* SOX17* and *TBX4. ABCC8* and *KDR* were added to the diagnostic genes at a later timepoint and were only tested for Family 3–5. Of the eight families without pathogenic variants identified by gene panel sequencing, five agreed to WES.

### Multiplex ligation-dependent probe amplification

To discover large deletions or duplications in the three genes *BMPR2*,* ACVRL1* and *ENG,* multiplex ligation-dependent probe amplification was performed (P093-C2, MRC-Holland, Netherlands).

### Whole exome sequencing

WES of nine patients and four healthy relatives belonging to 5 distinct HPAH families was performed on the NovaSeq 6000 (Illumina, USA). Exome libraries were prepared with the v.8 SureSelect Human All Exon kit (Agilent Technologies, USA). An average coverage of 300x was obtained with 96% of regions of coverage ≥ 20x. Trimming of the raw-fast-reads was done with next generation sequencing molecular barcode script (NextGen Toolkit v3.0.6, Agilent Genomics, USA). Mapping was performed with the alignment program hierarchical indexing for spliced alignment of transcripts 2 (HISAT2; https://daehwankimlab.github.io/hisat2) to the Genome Reference Consortium Human Build (version GRCh38/hg38; https://www.ncbi.nlm.nih.gov/datasets/genome/GCF_000001405.26)^12^. Variants were called applying deepvariant v.1.4.0 (google, USA; https://github.com/google/deepvariant) and annotated with Ensembl Variant Effect Predictor (v.107, EMBL-EBI, UK; https://www.ensembl.org/info/docs/tools/vep/index.html).

Resulting variants were filtered by firstly retaining only high-quality variants with minor allele frequency ≤ 0.0001, a total allele count of ≤ 5 in the genome Aggregation Database (gnomAD v3.1.2; https://gnomad.broadinstitute.org) and ≤ 5 in the inhouse database. Secondly, variants showing possible functional consequences were kept (nonsense, frameshift, non-frameshift indels, splice site and missense variants). Promoter variants were only investigated for *BMPR2*. In contrast, synonymous variants, intronic, intergenic, non-coding transcript variants were excluded. Rare missense variants were kept with a score of the in silico prediction program combined annotation dependent depletion (CADD; https://cadd.gs.washington.edu/snv) ≥15^13^ and splice site variants were kept if the deep learning-based tool SpliceAI score was ≥ 0.5^14^ (https://spliceailookup.broadinstitute.org). A detailed filtering process is provided in a supplementary file (Table S1), which narrowed down the number of rare variants of potential interest to around 30–120 per family. Candidate variants were visualized and quality checked on the Integrative Genomics Viewer (https://igv.org).

### Variant characterization

Gene disease association was investigated by databases GeneCards^15^ (https://www.genecards.org), Online Mendelian Inheritance in Man (OMIM, John Hopkins University, USA; https://www.omim.org/) and Human Gene Mutation Database professional (HGMD, version 2020.3, Qiagen, Germany; https://apps.ingenuity.com/ingsso/login). Molecular function of the candidate genes was examined on Uniprot (https://www.uniprot.org) and gene related pathways were sought on Open Targets Platform^16^ (https://platform.opentargets.org). Variants were characterized according to American College of Medical Genetics and Genomics (ACMG) and Association for Molecular Pathology (AMP) guidelines^17^ adapted for *BMPR2*^18^. Bayesian point based scoring system was applied to score variant criteria as supporting (± 1), moderate (± 2), strong (± 4) or very strong (± 8)^19^. Positive points indicate pathogenicity and negative points benignity. Pathogenic very strong criterion (PVS1, score + 8) was applied for predicted null variants, and pathogenic moderate criterion in supporting strength (PM2_supporting, score + 1) was used if variant population frequency was < 0.01%. Furthermore, pathogenic supporting (PP3, score + 1) or benign supporting (BP4, score − 1) criteria was employed for in silico predictions. Missense variants were considered pathogenic with a rare exome variant ensemble learner (REVEL; e.g. implemented in https://varsome.com/about/resources/germline-implementation/) score ≥ 0.75 or benign ≤ 0.25^20^. The identified variants were eventually classified as pathogenic (≥ 10), likely pathogenic (6 to 9), variants of uncertain significance (− 1 to 5), likely benign (− 2 to − 6) or benign (≤ − 7).

### RNA sequencing

Whole blood samples were collected with PaxGene collection tubes (BD, Germany). RNA was extracted using the PAXgene Blood RNA Kit (Qiagen, Germany). Library preparation was done using ribosomal RNA depletion with NEB Globin Depletion Kit (New England Biolabs, USA). Paired-end bulk sequencing of RNA with a 150-cycle kit (Illumina, USA) was performed on the NextSeq 550 (Illumina, USA). The resulting FASTQ files for forward and reverse reads for each patient were subjected to quality control using FastQC (https://www.bioinformatics.babraham.ac.uk/projects/fastqc) to assess read quality, GC content, and adaptor contamination. Low-quality bases and adaptor sequences were trimmed using Trimmomatic (https://github.com/usadellab/Trimmomatic), and post-trimming quality was reassessed with FastQC to ensure high-quality data. Cleaned reads were then analyzed for aberrant transcripts using the software Salmon (COMBINE-lab; https://github.com/COMBINE-lab/salmon) at transcript level^21^. A Log 2-fold change greater than 1 or less than − 1, combined with an adjusted p-value < 0.05 was considered significantly up or downregulated.

### Immunohistochemistry

Tissue from explanted lungs from the deceased patient of Family 5 (II:1) was sampled at autopsy. It was embedded in paraffin, cut in 5 μm slices and stained with hematoxylin-eosin-saffron staining as previously described^22^. For visualization conventional light microscopy was used and characterized by a pathologist experienced in PH (PD).

### Questionnaire

We designed a questionnaire for HPAH patients and healthy relatives to identify previous toxin and drug exposures to any substances listed as definite or possible associated with PAH in the ESC/ERS PH guidelines^1^ and extended these by agents and further possible triggering mechanisms identified in literature^23–26^. Substances were queried by generic and product name from the relevant category including agents affecting serotonin metabolism (anorexigens and antidepressants), interferons, antiviral drugs, tyrosine kinase inhibitors, immunosuppressants. The questionnaire also included less common, yet previously established, risk factors associated with PAH outbreaks, such as toxic rapeseed oil, L-tryptophan and Chinese herb Qing-Dai (induribin)^9,27,28^. We further collected information regarding the use of herbal preparation (e.g. St John’s Wort), chemical agents (e.g. trichloroethylene), opioids, chemotherapeutic agents and exposure to toxic dust (e.g. asbestos). Additionally, to obtain a comprehensive medical history of patients, factors addressing lifestyle choices, such as smoking status, hormone replacement therapy, and long-term residence at high altitude were investigated. Females were asked to provide information about time and number of pregnancies. This questionnaire was given to the patients and their healthy relatives of all five HPAH families.

## Results

Out of 47 families with HPAH, 39 carried (likely) pathogenic variants in a known PAH gene as reported previously^5^. Of the eight remaining families, five agreed to whole exome sequencing in 13 family members and to fill out a detailed exposure questionnaire. Index patients of two families were already deceased before being able to provide informed consent for this study and in one family both index patients were still minors. Detailed variant analysis revealed potentially PAH related variants in three of the five HPAH families, while no exposure to PAH associated drugs or toxins was recorded in these families. None of the family pedigrees revealed consanguinity. In contrast, in the remaining two families, an environmental exposure was identified as a likely trigger of the disease. None of the families showed large exon or gene deletions or duplications in the genes *BMPR2*, *ACVLR1* or *ENG*.

### Potential heritable genetic cause in three families

#### Family 1

**Fig. 1:**
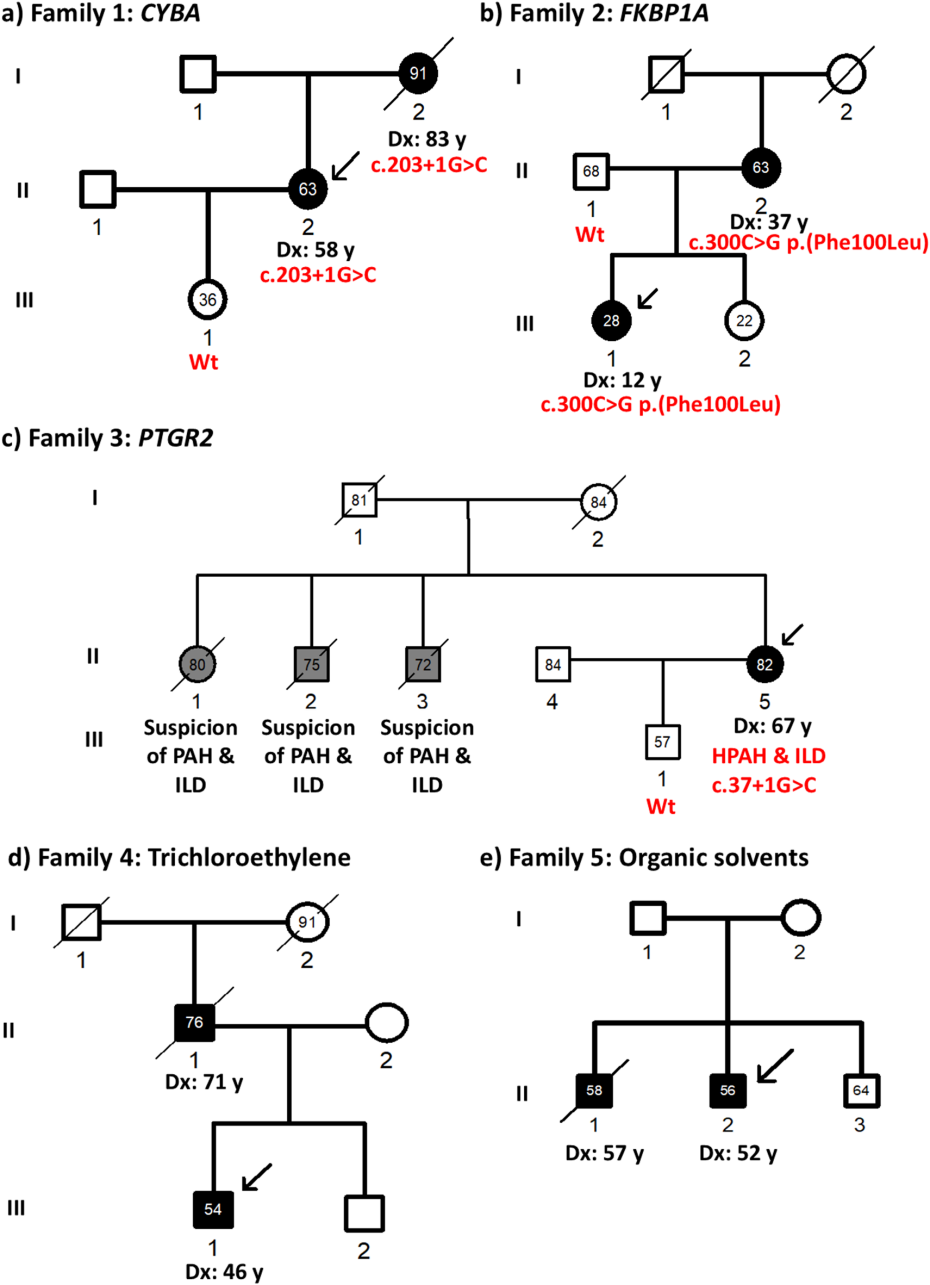
Pedigrees of all five families. (**a**) Affected mother (I:2) and daughter (II:2) in Family 1 carried the likely pathogenic splice site variant c.203 + 1G > C p.(?) in *CYBA* (cytochrome B-245 alpha chain). The variant was absent in the healthy granddaughter (III:1). (**b**) In Family 2, a predicted deleterious missense variant c.300 C > G p.(Phe100Leu) in *FKBP1A* (FKBP prolyl isomerase 1 A) was found in the index patient (III:1) and her mother (II:2). The healthy father had the wild type *FKBP1A* gene. (**c**) Family 3 was heterozygous for a likely pathogenic splice site variant c.37 + 1G > C p.(?) in *PTGR2* (prostaglandin reductase 2). The variant was only found in the affected mother (II:5) and not in her healthy son (III:1). No DNA samples were available from any other family members for segregation analysis. (**d**) Both the index patient (III:1) and his affected father (II:1) in Family 4 worked in different companies with exposure to trichloroethylene and both reported longer intake of the opioid tramadol. (**e**) Family 5 shows two affected brothers who worked in a paint factory. Both were exposed to toxic dust and different organic solvents in contrast to their healthy brother (II:3). Description of symbols: Filled: pulmonary arterial hypertension (PAH) confirmed with right heart catheterization; grey: suspicion of PAH with no medical records available; empty: healthy relatives; squares: males; circles: females. Dx: age at diagnosis, numbers within the symbol represent current age or age at death, deceased individuals are shown with a slash, arrows indicate the index patient.

In Family 1, mother and daughter were diagnosed with HPAH (Fig. 1A). The affected mother (I:2) was initially diagnosed with PAH associated with an antinuclear antibody positive undifferentiated connective tissue disease (CTD) with Raynaud’s syndrome and facial telangiectasias at an age of 83 years. CTD and PAH diagnoses were made simultaneously after a rapidly progressing dyspnea within weeks. Her daughter (II:2) was diagnosed with severe PAH at the age of 58 years. Considering the diagnosis of her mother, both were classified as HPAH. The daughter had a mean pulmonary arterial pressure (mPAP) of 65 mmHg and a pulmonary vascular resistance (PVR) of 10 Wood units (WU). The daughter showed no signs of CTD (Table 1).

**Table 1:** Clinical parameters of the five investigated families.

Families*	Genetic cause					Environmental exposure			
	Family 1		Family 2		Family 3	Family 4		Family 5	
Individuals	1:2	II:2	II:2	III:1	II:5	II:1	III:1	II:1	II:2
Sex	F	F	F	F	F	M	M	M	M
Age of diagnosis (years)	83	58	37	12	67	71	46	57	52
Diagnosis category	CTD-APAH DD HPAH	HPAH	HPAH	HPAH	HPAH & ILD	DPAH	DPAH	DPAH DD PVOD	DPAH DD PVOD
mPAP (mmHg)	39	65	66	38	25	31	27	41	36
PAWP (mmHg)	10	12	5	13	12	9	14	9	12
PVR (WU)	8.2	9.9	12.4	4.5	2.2	6.2	1.6	9.2	3.5
CO (l/min)	3.5	5.3	5.1	5.6	6.5	3.5	6.3	3.5	6.9
CI (l/min/m^2^)	2.1	2.5	2.6	3.3	3.1	2.1	2.5	3.4	x
6MWD (m)	240	301	462	560	615	406	414	300	421
WHO functional class	III	x	II-III	I	II-III	x	II	III	III
NT-proBNP (ng/l)	1194	188	224 (BNP)	82 (BNP)	179	89	18	34	711
FEV1 predicted (%)	76	63	82	103	139	104	82	70	84
DLCO predicted (%)	54	46	88	73	80	20	61	34	57
Blood gas analysis									
PO_2_ (mmHg) %	83.4	57	81.2	26	78.4	59.6	126	71.3	56
PCO_2_ (mmHg) %	26.6	41	28.5	47.2	33.9	68.2	36	30.1	29
SaO_2_%	97.2	90	96.8	98	97	88.2	97.5	95.9	91
Current therapy	Ambrisentan, Tadalafil	Tadalafil, Macitentan	Sildenafil, Macitentan	Sildenafil, Ambrisentan, Sotatercept	Sildenafil	Sildenafil, Ambrisentan	Macitentan	Tadalafil	Tadalafil, Ambrisentan
Findings	*CYBA*	*CYBA*	*FKBP1A*	*FKBP1A*	*PTGR2*	Trichloroethylene, asbestos, tramadol	Trichloroethylene, asbestos, tramadol	Organic solvents	Organic solvents

*Clinical data of patients corresponds to time of diagnosis.

CTD-APAH, connective tissue disease associated pulmonary arterial hypertension; *CYBA*, cytochrome B-245 alpha chain; CO, cardiac output; CI, cardiac index; DD, differential diagnosis; DLCO, diffusing capacity of the lungs for carbon monoxide; DPAH, drug or toxin associated pulmonary arterial hypertension; *FKBP1A*, FK506 binding protein 1 A; FEV1, forced expiratory volume in the first second; HPAH, heritable pulmonary arterial hypertension; ILD, interstitial lung disease; mPAP, mean pulmonary arterial pressure; 6MWD, 6-minute walking distance; NT-proBNP, N-terminal pro-brain natriuretic peptide; PAWP, pulmonary arterial wedge pressure; *PTGR2*, prostaglandin reductase 2; PVR, pulmonary vascular resistance; WU, Wood units; PVOD, pulmonary veno-occlusive disease; PO_2_, partial pressure of oxygen; PCO_2_, partial pressure of carbon dioxide; SaO_2_, arterial oxygen saturation; WHO, World Health Organization.

In addition, both mother and daughter were reported to have hypothyroidism as well as moderate hypoxemia. The mother had moderately reduced DLCO of 54% and the daughter had a DLCO of 46%. They were treated with double combination PAH therapy (Table 1). Mother and daughter carried a heterozygous, likely pathogenic, not yet described splice site variant c.203 + 1G > C p.(?) in the gene *CYBA* (cytochrome B-245 alpha chain) leading to a loss of the donor splice site in exon 3 of 5. Transcriptome data revealed a Log 2-fold downregulation of 1.3 (2.5 x reduction) for the canonical 195 amino acids encoding *CYBA* transcript (ENST00000261623) with 9 exons. Instead two non-coding transcripts (ENST00000562209.1 and ENST00000566534.5) were both upregulated with a Log 2-fold change of 1.9 (3.7 x upregulation). In addition, a longer coding transcript of 254 amino acids was identified with unclear functional implications (ENST00000696161.1). The variant was absent in both gnomAD v2.1.1 and v4.1.0 databases (Table 2). The *CYBA* gene encodes a catalytic component of nicotinamide adenine dinucleotide phosphate (NADPH) oxidase responsible for superoxide production.

**Table 2:** New candidate genes identified in three likely HPAH families.

Families	Genes	Variant consequence	HGVS nomenclature rsID if available	Molecular function	Associated diseases (OMIM)	Association to PAH	gnomAD	In silico predictions	ACMG class and criteria
Family 1	*CYBA*	Heterozygous splice donor site loss	NM_000101.4: c.203 + 1G > C p.(?) Intron: 3/5	Oxidoreductase	Chronic granulomatous disease 4 (AR) OMIM: 233690	− NADPH oxidase complex − Reactive oxygen species biosynthetic process − Smooth muscle hypertrophy	v4.1.0 ^b^: absent; v2.1.1 ^c^: absent	SpliceAI: 0.98	**LP** ^a^ (Score: +9) PVS1: +8 PM2_Supp: +1
	*NPR2*	Heterozygous missense	NM_003995.4: c.934C > T p.(Arg312Cys) Exon: 3/22 rsID: rs766889818	Guanylate cyclase activity	Acromesomelic dysplasia (AR) OMIM: 602875 Epiphyseal chondro-dysplasia, Miura type (AD) OMIM: 615923 Short stature with nonspecific skeletal abnormalities (AD) OMIM: 616255	− cGMP biosynthetic process − Blood vessels remodeling	v4.1.0 ^b﻿:^ 0.000002; v2.1.1 ^c^: absent	REVEL: 0.558 CADD: 25.0	**VUS** ^a^ (Score + 1) PM2_Supp: +1
Family 2	*FKBP1A*	Heterozygous missense	NM_000801.5: c.300C > G p. (Phe100Leu) Exon: 4/5	Peptidyl-prolyl cis-trans isomerase activity	-	− Type 1 transforming growth factor beta binding − FK506 binding − Activin receptor binding	v4.1.0 ^b^: absent; v2.1.1 ^c^: absent	REVEL: 0.73 CADD: 23.5	**VUS** (Score: +1) PM2_Supp: +1
Family 3	*PTGR2*	Heterozygous splice donor site loss	NM_001146154.2: c.37 + 1G > C p.(?) Intron: 2/9 rsID: rs145655340	13-prostaglandin reductase activity	-	Prostaglandin metabolic process	v4.1.0 ^b﻿:^ 0.000021; v2.1.1 ^c^: absent	SpliceAI: 0.99	**LP** (Score: +9) PVS1: +8 PM2_Supp: +1

^a^OMIM reported disease association (chronic granulomatous disease 4/acromesomelic dysplasia) due to the detected variant is not expected because of the autosomal recessive inheritance pattern and the detection of only a single variant within the gene; ^b^total minor allele frequency in gnomAD v4.1.0; ^c^ highest allele frequency in subpopulations of controls in gnomAD v2.1.1 with at least 1000 alleles.

ACMG, American College of Medical Genetics and Genomics; AD, autosomal dominant; AR, autosomal recessive, *CYBA*, cytochrome B-245 alpha chain; *FKBP1A*, FK506 binding protein 1 A; gnomAD, genome Aggregation Database; OMIM, Online Mendelian Inheritance in Man; *PTGR2*, prostaglandin reductase 2; rsID, reference single nucleotide polymorphism identification.

ACMG class: LP: likely pathogenic, VUS: variant of uncertain significance

ACMG criteria: PM2_supp: pathogenic moderate criterion scored supporting if population frequency was < 0.01% among gnomAD controls (+ 1); PP3: pathogenic supporting criterion for in silico predictions suggesting a deleterious impact (+ 1); PVS1: pathogenic very strong criterion for predicted null variants (+ 8).

Prediction programs: REVEL: rare exome variant ensemble learner with a score of ≥ 0.75 (pathogenic), 0.70−0.25 (uncertain) and ≤ 0.20 (benign); CADD: combined annotation dependent depletion with a score of ≥ 20 (pathogenic), 20 − 10 (uncertain) and ≤ 10 (benign); Splice AI with > 0.8 (pathogenic).

In addition to *CYBA*, we identified in both patients the rare, heterozygous, missense variant c.934C > T p.(Arg312Cys) in the gene *NPR2* (natriuretic peptide receptor 2, 0.0002% allele frequency in gnomAD v.4.1.0) (Table 2). The variant was characterized as a variant of uncertain significance (VUS). NPR2 has guanylyl cyclase activity to convert intracellular guanosine triphosphate to cyclic guanosine monophosphate (cGMP) relevant for vasodilation^29^. Both variants were absent in the to date healthy granddaughter (III:1) (Fig. 1A). The exposure questionnaire revealed no environmental toxins or drug intake potentially triggering PAH development in Family 1 (Table 3).

**Table 3:** Toxin and drug exposure in investigated families with PAH.

Families	Family 1			Family 2		Family 3		Family 4		Family 5		
Individuals	1:2	II:2	III:1	II:2	III:1	II:5	III:1	II:1	III:1	II:1	II:2	II:3
Sex	F	F	F	F	F	F	M	M	M	M	M	M
Age at diagnosis (years)	83	58	Healthy	37	12	67	Healthy	71	46	57	52	Healthy
Chemical/substance exposure												
Trichloroethylene	x	x	x	x	x	x	x	~Age: 46–56 y	Age: 16–23 y	x	x	x
Asbestos dust exposure	x	x	x	x	x	x	x	~Age: 46–56 y	Age: 23–26 y	x	x	x
Exposure to organic solvents	x	x	x	x	x	x	x	x	x	~Age: 28–55 y	Age: 29–52 y	x
Drug exposure												
Opioids (e.g. tramadol)	x	x	x	x	x	x	x	Age: 52 y	Since age 42 y	x	x	x
Cancer medications/immunosuppressants	x	x	x	x	x	Tamoxifen: Age 71–79 y	x	x	x	x	x	x
Antidepressants (sertotonin uptake inhibitors)	x	x	x	x	Escitalopram: Age 21 y	x	x	x	Escitalopram: Age: 45–51 y	x	x	x
Anorexigens/interferons/alkylating substances/antiviral medication/interferons/St. John’s Wort/Qing-Dai/toxic rapeseed oil/L-tryptophan	x	x	x	x	x	x	x	x	x	x	x	x
Life (style) choices												
Smoking history	x	x	x	x	x	x	x	80 pack- years	x	20 pack- years	Yes^1^	x
Long-term residence > 2500 m	x	x	x	x	x	x	x	x	x	x	x	x
Number of pregnancies, (age at each pregnancy in years)	3 (21, 23, 29)	2 (22, 27)	2 (28, 32)	3 (33, 35, 37*)	0	1 (26)	NA	NA	NA	NA	NA	NA

*Third pregnancy corresponds to the age of PAH onset; ^1^exact number of pack-years not available; F: female; M: male; NA: not applicable; x: no history or exposure identified; y: years.

#### Family 2

In Family 2, also mother and daughter were diagnosed with HPAH (Fig. 1B). The mother (II:2) was diagnosed aged 37 years shortly after her third and last pregnancy (Table 3). Her daughter (III:1) had childhood-onset HPAH at an age of 12 years. As a child she was started on sildenafil, amlodipine and at aged 15 years she was enrolled in a study for children receiving ambrisentan. As an adult with 26 years, she participated in the phase III study STELLAR for placebo vs. subcutaneous sotatercept and is currently receiving open-label sotatercept in the SOTERIA study (Table 1) in addition to sildenafil and ambrisentan.

Both mother and daughter carried a within this study newly described heterozygous missense variant c.300C > G p.(Phe100Leu) in the gene *FKBP1A* (FK506 binding protein 1 A), which was absent in the healthy father (II:1, Fig. 1B). The variant was absent in both gnomAD v2.1.1 and gnomAD v4.1.0 and predicted to be likely pathogenic with a REVEL score of 0.73 (≥ 0.75 pathogenic) and CADD score of 23.5 (≥ 20 pathogenic). Due to the gene’s involvement in TGF-β signaling, and its co-segregation with the disease, we considered it as a possible cause of PAH in this family. By ACMG criteria the variant was conservatively classified as a VUS (Table 2). No environmental exposure to toxins or drugs as potential external causes were reported by the members of Family 2 (Table 3).

### Family 3

The index patient (II:5) was diagnosed with mild PAH at the age of 67 years and 13 years later with mild interstitial lung disease (ILD) (Table 1; Fig. 1C). In addition, the index patient was also diagnosed with hypercholesterolemia and hyperlipidemia confirmed by high levels of cholesterol [261 mg/dl (reference values 0–200 mg/dl)] and elevated triglycerides levels [354 mg/dl (reference values 0–150 mg/dl)]. Three deceased siblings of the index patient had both reported ILD and a suspicion of PAH (Fig. 1C). No medical records or DNA samples were available to obtain information on the exact cause of death or to confirm a HPAH diagnosis of the siblings. Therefore, a co-segregation analysis could not be performed.

We detected the heterozygous splice site variant c.37 + 1G > C p.(?) in the gene *PTGR2* (prostaglandin reductase 2) in the second of nine introns (Table 2) in the index patient but not in the healthy son (III:1). A loss of the splice donor site of intron 2 was predicted to either lead to intron 2 retention or exon skipping of exon 2 (37 coding base pairs) resulting in a frameshift and premature stop codon with subsequent nonsense mediated decay or an alternative RNA isoform. The variant was absent among controls in gnomAD v2.1.1 and present at very low frequency (0.0021%) in gnomAD v4.1.0. The transcriptome analysis of *PTGR2* isoforms in the affected individual revealed a complete loss of the main transcript (MANE: ENST00000555661.6) in contrast to the healthy son without the identified variant. However, an alternative transcript with the same number of amino acids was upregulated in the patient (ENST00000267568.8). It is unclear whether the upregulation was sufficient to fully compensate the lost main transcript and whether expression was achieved in all tissues. Overall, the variant was classified as likely pathogenic according to ACMG criteria (Table 2). *PTGR2* is involved in prostaglandin metabolism^30^. No additional exposure to environmental toxins or drugs associated with PAH development could be identified in the index patient of Family 3 (Table 3).

Variant filtering steps in all five HPAH families are enlisted in Table S1 showing a reduction of variants from up to 37.000 to around 30–110 per family. Further rare variants of uncertain significance with hypothetical relevance to vascular pathways in PAH are listed in Table S2.

### Families with occupational exposure

#### Family 4

The genetic work-up revealed no (likely) pathogenic variants possibly associated with PAH in this family. Instead of a potential genetic disease cause, we identified a common, independent, occupational long-term exposure to trichloroethylene and asbestos dust in both affected family members, in the father (II:1) and his son (III:1) (Fig. 1D; Table 3). The father worked as a mechanic in a company and was diagnosed with PAH at 71 years of age. He had also suffered from mild pulmonary fibrosis as a comorbidity. Computed tomography (CT) scan showed slightly basal bilateral fibrosis. While trichloroethylene has been associated with PVOD before^1^ no clear evidence of PVOD was identified in the CT scan of the patient. The father also had a smoking history and he consumed two packs of cigarettes per day for over approximately 40 years, equivalent to around 80 pack-years (Table 3).

The son was diagnosed with PAH at an age of 46 years. He worked as a radiational and environmental protection technician in a company where he was responsible for the disposal of hazardous waste. He had exposure to trichloroethylene as well as inhaled asbestos dust, plutonium, radioactive isotopes strontium-90, cesium-134, cobalt-60, albeit while wearing protective gear. The son mentioned no history of smoking (Table 3).

His CT scan showed no evidence of PVOD neither. In addition, the son reported to have taken the opioid tramadol for more than 10 years prior to PAH onset and the father for the duration of 1 year albeit 19 years prior to PAH manifestation. Tramadol has also been reported previously as a potential risk factor for PAH^31,32^. Taken together, most likely long-term trichloroethylene exposure led to PAH onset in both affected family members with opioid intake and asbestos exposure as potential additional triggers, mimicking a hereditary phenotype in Family 4.

#### Family 5

Similarly, no likely pathogenic variants associated with PAH genes could be identified in both affected brothers (II:1 and II:2) in Family 5, but a common occupational toxic dust exposure. Both patients worked in the same paint factory and had exposure for more than 20 years to toxic dust containing several organic solvents, such as petroleum, white mineral oil and benzene compounds. The healthy third brother (II:3) did not work in the same factory and had no PAH (Fig. 1E; Table 3).

**Fig. 2:**
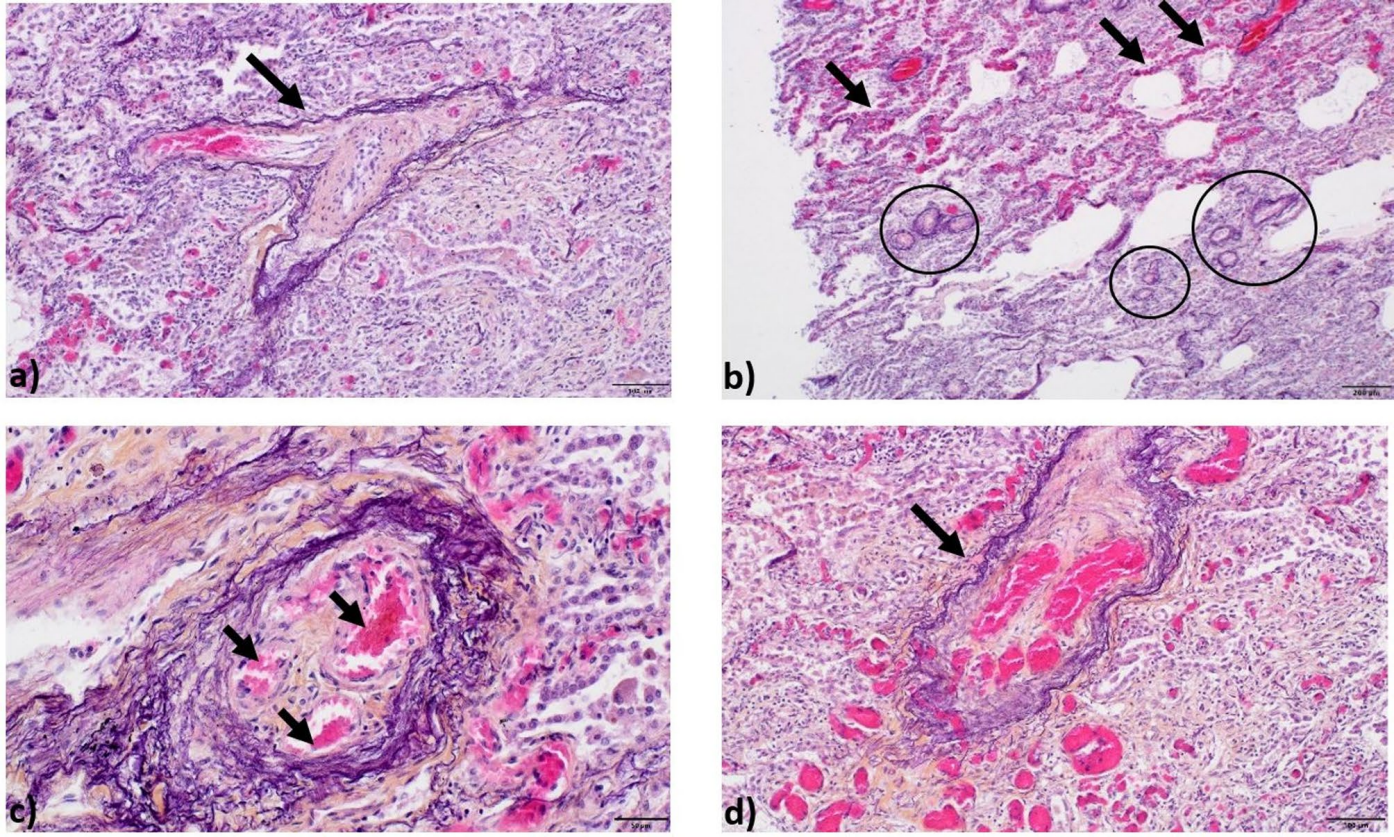
Histological confirmation of pulmonary veno-occlusive disease in individual II:1 of Family 5. Lung tissue was stained after autopsy with hematoxylin-eosin-saffron staining. Pulmonary veno-occlusive disease was characterized by (**a**) likely venous remodelling indicated by black arrow, scale bar = 100 μm, (**b**) pulmonary capillary hemangiomatosis and congestion (arrows), and microvascualr remodelling (circles), scale bar = 200 μm and (**c,d**) venous thrombosis shown by arrows, scale bar = 50 μm in Fig. 2c and 100 μm in Fig. 2d.

The brother (II:1) of the index patient was diagnosed with PAH with an unclear etiology and differential diagnosis of PVOD at the age of 57 years. He had a reduced DLCO of 34%. CT scan showed enlarged lymph nodes and thickened interlobular septa and ground glass opacities consolidating the suspicion of PVOD. The patient smoked 20 pack-years (Table 3). He died 1 year after the PAH/PVOD diagnosis. An autopsy was carried out after his death. The histopathological analysis of his lung tissue, which we have performed, confirmed an unusual form of PVOD with remodeling of the venules with an atypical amount of venous thrombosis and capillary neo-angiogenesis (Fig. 2). The index patient (II:2) was diagnosed with a less severe form of PVOD aged 52 years with a DLCO of 57%, mPAP of 36 mmHg, PVR of 3.5 WU and PAWP of 12 mmHg (Table 1). The CT revealed increased abdominal lymph nodes, and chest X-ray indicated possible pulmonary venous congestion. His exposure to paint dust was less intense than for his brother. The index patient also reported to have smoking history but the exact information on pack-years was not available (Table 3), he had then ceased smoking. The patient’s condition stabilized under double combination therapy and optimized diuretics. He is currently being evaluated for lung transplantation.

## Discussion

In this study we detected new, to the best of our knowledge not yet described rare missense and likely pathogenic variants in potentially novel candidate PAH genes in index patients and affected relatives of three HPAH families. To establish a definite gene-disease relationship further functional studies would however be required. The diagnosis of the two remaining PAH families with two affected members each was revised as not heritable. Instead, their disease was most likely due to environmental factors. Thus, in the genetic and clinical work-up it is essential not only to consider genetic causes but also any potential drug or toxin exposures as PAH triggers.

### Potential novel PAH candidate genes

In Family 1 a likely pathogenic heterozygous splice site variant in the gene *CYBA* was identified. *CYBA* encodes the protein p22 phagocyte oxidase (p22phox), which forms a membrane associated heterodimer complex with the NADPH oxidase and is essential for its catalytic activity^33^. NADPH oxidases have been reported as the major enzymatic source of reactive oxygen species (ROS) in the vasculature, particularly in generating superoxide anions (O_2_^−^) by transferring electrons from NADPH to molecular oxygen^34^. ROS in turn play a crucial role in pathophysiology of PH by causing endothelial dysfunction, altering vasoconstrictive response and pulmonary vascular remodeling^35^.

Mice with a homozygous *CYBA* knock out (p22phox^−/−^) under hypoxic conditions were protected against PH, while wild type mice in hypoxia developed pulmonary vascular remodeling and increased vasoconstriction^36^. In our study, we identified a heterozygous splice site variant that could have had a different effect than a homozygous variant. In addition, transcriptomic data analysis revealed that the *CYBA* canonical transcript was downregulated in Family 1, and another, longer coding transcript with unclear functional implication was identified. This suggests that another *CYBA* isoform was being used which is either maintaining the normal gene function or is potentially resulting in increased production of p22phox, thereby possibly enhancing the catalytic activity of NADPH oxidases and increased generation of ROS species. To clarify the effect of the different isoforms functional studies would have to be conducted which was beyond the scope of this study.

Both patients in Family 1 also had moderate hypoxemia that could lead to low blood oxygen levels. Zhang and colleagues^37^ identified that hypoxia stabilizes p22phox by reducing its ubiquitination and proteasomal degradation, leading to increased generation of ROS species^37^. P22phox also contributed to increased levels of hypoxia inducible factor 1-alpha and the downregulation of miR-140, resulting in increased cell proliferation, migration and angiogenesis^38^. This might offer another explanation for the potential pathomechanisms, how the identified *CYBA* variant could have derived PAH development in Family 1. However, functional data is mandatory to determine the true pathogenic effects.

In addition to *CYBA*, a heterozygous missense variant in the *NPR2* (natriuretic peptide receptor 2) gene was found in both the affected mother and her daughter. *NPR2* is expressed in vascular smooth muscle cells and induces cGMP production^39,40^ but has not yet described with vascular remodeling and/or the development of PH. PAH could therefore be a new phenotype associated with this gene which remains to be explored and validated in further studies.

In Family 2, both mother and daughter, who were diagnosed with HPAH carried a heterozygous missense variant in the *FKBP1A* (FK506-binding protein 1 A) gene. FKBP1A encodes a cis-trans prolyl isomerase that interacts with several intracellular signal transduction pathways including TGF-β type 1 receptors. FKBP1A binds to type 1 receptors and regulates BMP and TGF-β signaling by blocking the receptor activation in the absence of ligands^41^. The addition of tacrolimus (FK506), a therapeutic target and an immunosuppressant of FKBP1A, dissociates FKBP1A from the receptor^42^, thereby activating downstream signaling. Tacrolimus has therefore been tested in a randomized, placebo-controlled phase IIa trial in PAH, which demonstrated its safety and tolerability, albeit with only mild effects on hemodynamics^43^. The missense variant in *FKBP1A* may have disturbed the tightly balanced BMPR2 and TGF-β signaling. The variant was, however, only classified as a VUS and therefore requires further functional evidence to confirm or exclude any disease-causing potential.

In Family 3, a likely pathogenic heterozygous splice variant in the prostaglandin reductase 2 (*PTGR2)* gene could have led to PAH. It acts as an endogenous ligand for peroxisome proliferator activated receptor gamma (PPARγ)^30^. PPARγ is also abundantly expressed in pulmonary vascular endothelial and smooth muscle cells where it shows anti-proliferative and pro-apoptotic activities^44^. Hence, *PTGR2* and its substrates are involved in balancing the tissue homeostasis of proliferation and apoptosis. Upstream of the substrate of *PTGR2* gene, is the prostaglandin PGE2, which is also the basis for the vasodilator prostacyclin (PGI2)^45^. Thus, *PTGR2* is involved in one of the main natural vasodilatory pathways, which are one of the therapeutic avenues in PAH treatment. Nevertheless, PTGR2 is only a candidate gene and further PAH patients with (likely) pathogenic *PTGR2* variants are warranted to establish a gene-disease relationship.

Moreover, PTGR2 was shown to harbor transcription factor binding sites for the PAH gene *SOX17*^46^. *SOX17* is a well-established, definitive PAH gene^4^. An off-set downstream signaling of SOX17 may also contribute to PAH development. Thus overall, a malfunctioning of prostaglandin reductase 2 might have disturbed the pulmonary arterial vasculature.

In addition to PAH, the index patient in Family 3 (II:5) also reported to have a history of ILD, hyperlipidemia and hypercholesterolemia. Interestingly, PPARγ also has a prominent role in lipid metabolism and serves as a master regulator of adipogenesis and lipid metabolism^30^. Its inactivation can enhance TGF-β signaling, potentially leading to increased inflammation, epithelial mesenchymal transition and pulmonary fibrosis^47–49^. Dyslipidemia has also been linked to right ventricular dysfunction and may be associated with development and progression of ILD and PH^50,51^.

### Environmental triggers of PAH

For the remaining two families the detailed occupational history and comprehensive questionnaire revealed a common environmental exposure among family members with PAH making them appear to have a heritable disease. In Family 4, both father and son had exposure to trichloroethylene during their work. PVOD, systemic sclerosis, and ILD were reported in trichloroethylene exposed workers^52^. Montani and colleagues performed a case control study to evaluate the association between different occupation exposures in 33 PVOD and 65 PAH patients. They identified trichloroethylene exposure as a significant risk factor in 58% of the 24 PVOD patients with any occupational toxin exposure^53^. Interestingly, none of the PVOD patients in the study of Montani and colleagues with an intermediate or high trichloroethylene exposure carried predisposing pathogenic variants in the gene *EIF2AK4*. While PVOD is the predominant phenotype reported, very few PAH patients with trichloroethylene exposure have been identified to date^53,54^. The two PAH patients in Family 4 showed no signs of PVOD. Thus, together with the previous cases trichloroethylene should also be considered as a causative substance not only for PVOD but also for PAH.

In addition to the trichloroethylene exposure both patients reported intake of the opioid tramadol, which may also have contributed to PAH development^31,32^. Moreover, both had an occupational exposure to asbestos dust, usually leading to ILD but in rare cases also to PH^55,56^. Nevertheless, not identified genetic alterations in both father and son could have contributed or led to PAH development.

In Family 5, two siblings were diagnosed with PVOD. They were employed in a paint factory where they had a long-term exposure to different organic solvents including petroleum, white mineral oil, and benzene compounds. Exposure to organic solvents including benzene and petroleum solvents was previously shown to be significantly associated with a PVOD diagnosis^53^. Six PVOD patients with occupational paint exposure were reported by Montani et al.^53^ and an additional case was documented by Triantafyllidi et al.^57^. In addition to organic solvents, both brothers had a smoking history. Tobacco smoke also damages the endothelial barrier^58^, which coincides in many PVOD patients with previous organic compound exposure^53^ possibly potentiating the toxic effects.

Both had late onset of PVOD, around the age of 50 years while *EIF2AK4* carriers have an early disease onset around 25 years of age^59^. This supports our conclusion that the occupational exposure to organic solvents was the most likely cause of PVOD in both brothers of Family 5. Regardless, further undetected genetic or epigenetic factors may have been the true cause or an additional trigger for the disease in the two brothers.

Our findings highlight the need to employ a holistic approach in clinical settings to gather detailed information regarding environmental factors besides genetic testing, because environmental toxin exposure may accumulate within a family, which makes it appear as if it were a genetic disorder. In addition, external factors may also act as a second hit in patients who do carry a pathogenic variant.

Of note, the initial 39 HPAH patients with a pathogenetic variant in a previously known PAH gene had not received the exposure questionnaire. In future it will be handed out to all HPAH patients, also including those with identified pathogenic variants, to better understand the necessary “second hit” for disease manifestation.

### Limitations

While we tried to employ a comprehensive approach to identify the disease cause in the five families, identifying underlying functional mechanisms of the highlighted, potentially disease-causing variants were beyond the scope of this study. Additional co-segregation of identified variants would have strengthened the gene-disease relationship. However, in some families as in Family 3, further patients had already died rendering genetic testing impossible. Nevertheless, two of the three variants in potentially novel PAH candidate genes could be classified as likely pathogenic following ACMG guidelines even without co-segregation data. It is important to note that ACMG classification may be misleading and that even variants detected in healthy controls can be classified as (likely) pathogenic by the criteria. So far, there is no established gene-disease relationship for the novel, identified candidate genes in this study and PAH. Therefore, for the true interpretation of the pathogenicity of these variants, functional data, and large-scale disease datasets are mandatory, and the implication of these genes should be interpreted with great caution. Moreover, as large case-control analyses have not identified any of the genes of this study^60,61^ as having a significantly increased number of deleterious variants in patients vs. healthy controls, most likely only a small subset of PAH patients may be carriers of (likely) pathogenic variants in the highlighted genes. Once the majority of the more frequent PAH genes has been identified through large-scale studies most likely private genes, restricted to only a few PAH families, may offer an explanation for the hereditary cause of the disease in the remaining genetically unsolved PAH families.

Our focus was to identify rare variants in coding and splice regions. However, it has been previously reported that the polymorphisms or common variants in the regulatory and promoter region can also affect the expression of PAH related genes^62–66^. Regulatory and non-coding variants were only partially captured by our WES approach and assessing common variants was beyond the scope of this study. While the environmental toxin exposure seems plausible in two of the families, we cannot rule out an additional genetic predisposition as a second hit. Likewise, unreported environmental exposure cannot be excluded in patients with identified (likely) pathogenic variants. Moreover, a genome-wide copy number variation analysis and the investigation of aberrant DNA methylation may have yielded further molecular insights.

## Conclusions

In three families we could identify rare potentially relevant variants in three novel PAH candidate genes in multiple affected patients. The heterozygous canonical splice site variant in the *CYBA* gene could have increased ROS signaling and drive smooth muscle hypertrophy. The missense variant in the *FKBP1A* gene could potentially dysregulate TGF-β signaling. The heterozygous splice site variant in the *PTGR2* gene could interfere with prostacyclin metabolism, which is pharmaceutically addressed in PAH patients. In the two remaining families, toxic exposure at the workplace to trichloroethylene or paint dust exposure were the most likely cause of toxin induced PAH. Hence, it is essential to gather detailed information of HPAH and PVOD patients including genetic and environmental causes to identify the main disease cause and potential additional genetic or environmental second hits.

## Supplementary Information

Below is the link to the electronic supplementary material.

Supplementary Material 1

## Data Availability

The datasets generated and/or analyzed during the current study are available in the European Genome-phenome Archive (EGA) repository (https://ega-archive.org/datasets/EGAD50000001817) under the sample accession numbers EGAF50000433816 to EGAF50000433828. The exposure questionnaire is available upon personal request.
