# Supplementary material for: Heritable pulmonary arterial hypertension: new genetic findings and environmental triggers
Source: Sci Rep. 2026 Jan 29;16:4121. doi: 10.1038/s41598-025-34167-0 (PMC12858969; doi:10.1038/s41598-025-34167-0)
Supplement: Supplementary file 1 — Supplementary Material 1 [file 41598_2025_34167_MOESM1_ESM.docx]

**Heritable pulmonary arterial hypertension – new genetic findings and environmental triggers**

**Short title: HPAH genetics and environmental triggers**

Memoona Shaukat^1,2^, Ekkehard Grünig^1^, Simon Haas^1^, Jan Haas^3^, Mohammad Panahi^1^, Martin Granzow^2^, Tobias J. Lange^4,5^, Stefan Stadler^5^, Natascha Sommer^6^, Peter Dorfmüller^7^, Benjamin Meder^3,8^, Satenik Harutyunova^1^, Benjamin Egenlauf^1^, Panagiota Xanthouli^1,9^, Katrin Hinderhofer^2^, Christina A. Eichstaedt^1,2^*

^1^ Center for Pulmonary Hypertension, Thoraxklinik Heidelberg gGmbH at Heidelberg University Hospital and Translational Lung Research Center Heidelberg (TLRC), German Center for Lung Research (DZL), Heidelberg, Germany

^2^ Laboratory for Molecular Genetic Diagnostics, Institute of Human Genetics, Heidelberg University, Heidelberg, Germany

^3^ Institute for Cardiomyopathies Heidelberg (ICH) and Center for Cardiogenetics, Heidelberg University Hospital, and DZHK, Site Heidelberg/Mannheim, Heidelberg, Germany

^4^ Department of Pneumology, Kliniken Südostbayern AG, Kreisklinik Bad Reichenhall, Bad Reichenhall, Germany

^5^ Department of Internal Medicine II, University Medical Center Regensburg, Regensburg, Germany

^6^Medical and Policlinic II, Excellence Cluster Cardio-Pulmonary Institute (CPI), , Member of the German Center for Lung Research (DZL), Justus-Liebig University Giessen, Giessen, Germany

^7^ Institute for Lung Health (ILH), Giessen, University Hospital of Gießen and Marburg, Universities of Giessen and Marburg Lung Center (UGMLC), German Center for Lung Research (DZL), Gießen, Germany

^8^ Department of Internal Medicine III, Precision Digital Health, University of Heidelberg and Informatics for Life, and German Center for Cardiovascular Research (DZHK), Heidelberg, Germany

^9^ Division of Rheumatology, Department of Internal Medicine V: Hematology, Oncology and Rheumatology, University Hospital Heidelberg, Heidelberg, Germany

***Corresponding author:**

Prof. Dr. Christina A. Eichstaedt

Centre for Pulmonary Hypertension

Thoraxklinik Heidelberg at Heidelberg University Hospital,

Röntgenstraße 1

Heidelberg 69126

Germany

Phone: +49-6221-396-1221

Mail address: [christina.eichstaedt@med.uni-heidelberg.de](mailto:christina.eichstaedt@med.uni-heidelberg.de)

ORCID: 0000-0001-7288-8297

**Supplementary file**

This file contains detailed information about the filtering criteria applied for analyzing whole exome sequencing (WES) data (Table S1).

**Table S1: WES variants’ filtering process for Families 1 - 5**

| **Filters** | **Family 1** | **Family 2** | **Family 3** | **Family 4** | **Family 5** |
| --- | --- | --- | --- | --- | --- |
| Total number of variants at MAF ≤ 5% | 13,952 | 14,407 | 24,375 | 37,568 | 12,087 |
| Only present in affected individuals | 5,104 | 8,285 | 7,844 | 21,084 | 4,861 |
| Gtsum (≤ 5) | 2,118 | 3,709 | 3,828 | 4,462 | 2,195 |
| gnomAD_MAF (≤ 0.01%) | 63 | 132 | 228 | 136 | 52 |
| gnomAD_AC (≤ 5) | 58 | 123 | 216 | 119 | 52 |
| Excluded variants ^a^ | 29 | 59 | 104 | 57 | 22 |
| Remaining total variants | 29 | 64 | 112 | 62 | 30 |
| Remaining missense variants | 14 | 44 | 74 | 47 | 20 |
| Remaining missense (CADD ≥15) | 12 | 31 | 54 | 33 | 13 |
| Remaining splice site variants | 8 | 12 | 18 | 11 | 5 |
| Remaining frameshift | 4 | 0 | 9 | 1 | 2 |
| Remaining nonsense & stop-retained | 2 | 2 | 0 | 1 | 0 |
| Remaining 5’ UTR variants | 1 | 6 | 11 | 2 | 3 |

^a^ excluded variants were: synonymous, intronic, non-coding transcript exon variants, 3’ UTR variants, > 20 bp upstream or downstream gene variants or intergenic variants.

AC, allele count in gnomAD; CADD: combined annotation dependent depletion; gnomAD, Genome Aggregation Database; Gtsum, genotype sum of all sequenced individuals corresponding to in-house allele count; MAF, minor allele frequency in gnomAD; UTR, untranslated region

**Table S2: Further variants of uncertain significance with hypothetical relevance to vascular pathways in five HPAH families**

| **Families** | **Genes** | **Variant**  **type** | **HGVS nomenclature** | **Molecular function** | **Associated diseases (OMIM)** | **Association to PAH** | **gnomAD** | ***In silico* predictions** | **ACMG**  **class & criteria** |
| --- | --- | --- | --- | --- | --- | --- | --- | --- | --- |
| Family 1 | *GRK5* | Heterozygous  missense | NM_005308.3:  c.289A>C  p.(Lys97Gln)  Exon: 4/16 | Serine / threonine protein kinase | - | Wnt signaling pathway  Anti-apoptotic | v4.1.0 ^b^:  0.00000062  v2.1.1 ^c^: absent | REVEL: 0.113  CADD: 24.2 | **VUS**  (Score: 0)  PM2_supp: +1  BP4: -1 |
|  | *RNASEH2A* | Heterozygous Splice donor | NM_006397.3:  c.761+1G>A  p.(?)  Intron: 7/7 | RNA-DNA hybrid ribonuclease activity | Aicardi-Goutieres syndrome (AR)  MIM: 610333 | Aicardi-Goutieres syndrome can be associated with PH | v4.1.0 ^b^:  0.00000062  v2.1.1 ^c^:  absent | SpliceAI: 0.99 | **LP** ^a^  (Score: +9)  PVS1: +8  PM2_supp: +1 |
|  | *PARM1*  (also in healthy family member) | Heterozygous  missense | NM_015393.4:  c.832G>T  p.(Ala278Ser)  Exon: 3/4 | Regulates telomerase activity | - | Proliferation of pulmonary arterial smooth muscle cells | v4.1.0 ^b^:  0.0000081  v2.1.1 ^c^: 0.000026 | Revel: 0.164  CADD: 25.4 | **VUS**  (Score: 0)  PM2_supp: +1 |
| Family 2 | *IGFBP4* | Heterozygous  missense | NM_001552.3:  c.538C>T  p.(Arg180Trp)  Exon: 3/4 | Insulin-like growth factor I & II binding | - | Regulation of cell growth | v4.1.0 ^b^:  0.0000179  v2.1.1 ^c^:  0.0001038 | REVEL: 0.388  CADD: 27.6 | **VUS**  (Score: 0) |
|  | *MAP3K6* | Heterozygous  missense | NM_004672.5:  c.664C>T  p.(Arg222Cys)  Exon: 4/29 | Serine / threonine protein kinase | - | Regulation of vascular endothelial growth factor expression | v4.1.0 ^b^:  0.000015  v2.1.1 ^c^: 0.0000704 | Revel: 0.067  CADD: 21 | **VUS**  (Score: 0)  PM2_supp: +1  BP4: -1 |
| Family 3 | *GCN1* | Heterozygous  missense | NM_006836.2: c.866C>T  p.(Thr289Met)  Exon: 10/58 | Protein kinase regulator activity | - | GCN2/ EIF2AK4-mediated signaling | v4.1.0 ^b^:  0.00001241  v2.1.1 ^c^:  0.00014 | Revel: 0.12  CADD: 23.7 | **VUS**  (Score: -1)  BP4: -1 |
|  | *CTNNA3* | Heterozygous  missense | NM_013266.4: c.138C>A  p.(Ser46Arg)  Exon: 3/18 | Actin filament & beta-catenin binding | Arrhythmo-genic right ventricular dysplasia 13 (AD)  MIM: 615616 | Regulation of ventricular cardiac muscle cell action potential | v4.1.0 ^b^:  0.00000186  v2.1.1 ^c^:  absent | REVEL: 0.015  CADD: 17.0 | **VUS**  (Score: 0)  PM2_supp: +1  BP4: -1 |
|  | *HIF1AN*  (also in healthy family member) | Heterozygous  missense | NM_017902.3: c.592G>A  p.(Ala198Thr)  Exon: 4/8 | - Notch binding - Oxygen sensor activity |  | Negative regulation of NOTCH signaling pathway  Vasculo-  genesis | v4.1.0 ^b^:  0.00000124  v2.1.1 ^c^:  absent | REVEL: 0.278  CADD: 23.5 | **VUS**  (Score: +1)  PM2_supp: +1 |
| Family 4 | *ITGAV* | Heterozygous  missense | NM_002210.5: c.1708G>A  p.(Ala570Thr)  Exon: 17/30 | - G-protein coupled receptor - Host cell receptor for virus entry |  | Transforming growth factor beta binding  Vasculoge-nesis  Angiogenesis | v4.1.0 ^b^:  0.00001940  v2.1.1 ^c^:  0.00014 | REVEL: 0.24  CADD: 23.9 | **VUS**  (Score: -1)  BP4: -1 |
| Family 5 | *DCHS1* | Heterozygous  missense | NM_003737.4: c.2018T>C  p.(Val673Ala)  Exon: 4/21 | Cadherin binding | Van Maldergem syndrome 1 (AR)  MIM: 601390  Mitral valve prolapse 2 (AD)  MIM: 601390 | Mitral valve formation | v4.1.0 ^b^:  0.00000062  v2.1.1 ^c^:  absent | REVEL: 0.507  CADD: 25.3 | **VUS** ^a^  (Score: -1)  PM2_supp: +1 |
|  | *MEGF8* | Heterozygous  missense | NM_001271938.2: c.8171C>T  p.(Ala2724Val)  Exon: 42/42 | Calcium ion binding | Carpenter syndrome 2 (AR)  MIM: 614976 | BMP signaling pathway  Coronary vascular development | v4.1.0 ^b^:  0.00000620  v2.1.1 ^c^:  absent | Revel: 0.078  CADD: 17.8 | **VUS** ^a^  (Score: 0)  PM2_supp: +1  BP4: -1 |

^a^ OMIM reported disease association (Aicardi-Goutieres syndrome / Van Maldergem syndrome 1 / Carpenter syndrome 2) due to the detected variant is not expected because of the autosomal recessive inheritance pattern and the detection of only a single variant within the gene; ^b^ total MAF in gnomAD v4.1.0; ^c^ highest MAF in subpopulations in controls in gnomAD v2.1.1 with at least 1000 alleles

AD: autosomal dominant; AR: autosomal recessive; ACMG: American College of Medical Genetics and Genomics; cGMP: cyclic guanosine monophosphate; *CTNNA3*: catenin alpha 3; *DCHS1:* dachsous cadherin-related 1; *GCN1:* GCN1 activator of EIF2AK4; *GRK5*: G protein-coupled receptor kinase 5; gnomAD: genome aggregation database; HGVS: human genome variation society; *HIF1AN*: hypoxia inducible factor 1 subunit alpha inhibitor; *IGFBP4:* insulin like growth factor binding protein 4; *ITGAV*: integrin subunit alpha V; MAF: minor allele frequency ; *MAP3K6:* mitogen-activated protein kinase kinase kinase 6; *MEGF8*: multiple EGF like domains 8; *NPR2*: natriuretic peptide receptor 2; OMIM: Online Mendelian Inheritance in Man; *PARM1*: prostate androgen-regulated mucin-like protein 1; *RNASEH2A:* ribonuclease H2 subunit A

**ACMG class:** LP: likely pathogenic; PVS1: pathogenic very strong; VUS: variant of uncertain significance

**ACMG criteria:** BP4:benign supporting criterion for *in silico* predictions suggesting no impact on gene product (-1); PM2_supp: pathogenic moderate criterion scored supporting if population frequency is <0.01% among gnomAD controls (+1); PVS1: pathogenic very strong criterion for predicted null variants (+8).

**Prediction programs:** REVEL: rare exome variant ensemble learner with a score of ≥0.70 (pathogenic), 0.75-0.25 (uncertain) and ≤0.20 (benign); CADD: combined annotation dependent depletion with a score of ≥20 (pathogenic), 20-10 (uncertain) and ≤10 (benign); SpliceAI with >0.8 (pathogenic)
